# Network pharmacology integrated molecular dynamics reveals the bioactive compounds and potential targets of *Tinospora crispa* Linn. as insulin sensitizer

**DOI:** 10.1371/journal.pone.0251837

**Published:** 2022-06-23

**Authors:** Ummu Mastna Zuhri, Erni Hernawati Purwaningsih, Fadilah Fadilah, Nancy Dewi Yuliana

**Affiliations:** 1 Doctoral Program in Biomedical Science, Faculty of Medicine, Universitas Indonesia, Jakarta, Indonesia; 2 Department of Medical Pharmacy, Faculty of Medicine, Universitas Indonesia, Jakarta, Indonesia; 3 Department of Medical Chemistry, Faculty of Medicine, Universitas Indonesia, Jakarta, Indonesia; 4 Bioinformatics Core Facilities, IMERI Faculty of Medicine, Universitas Indonesia, Jakarta, Indonesia; 5 Department of Food Science and Technology, Bogor Agricultural University, Bogor, Indonesia; Government College University Faisalabad, PAKISTAN

## Abstract

Insulin resistance is a metabolic disorder characterized by the decreased response to insulin in muscle, liver, and adipose cells. This condition remains a complex phenomenon that involves several genetic defects and environmental stresses. In the present study, we investigated the mechanism of known phytochemical constituents of *Tinospora crispa* and its interaction with insulin-resistant target proteins by using network pharmacology, molecular docking, and molecular dynamics (MD) simulation. Tinoscorside A, Makisterone C, Borapetoside A and B, and β sitosterol consider the main phytoconstituents of *Tinospora crispa* by its binding with active sites of main protein targets of insulin resistance potential therapy. Moreover, Tinoscorside A was revealed from the docking analysis as the ligand that binds most strongly to the target protein, PI3K. This finding was strengthened by the results of MD simulation, which stated that the conformational stability of the ligand-protein complex was achieved at 15 ns and the formation of hydrogen bonds at the active site. In conclusion, *Tinospora crispa* is one of the promising therapeutic agent in type 2 diabetes mellitus management. Regulation in glucose homeostasis, adipolysis, cell proliferation, and antiapoptosis are predicted to be the critical mechanism of *Tinospora crispa* as an insulin sensitizer.

## Introduction

Insulin resistance is a metabolic disorder characterized by the decreased response to insulin in muscle, liver, and adipose cells. The normal insulin levels are unable to control glucose, lipids, and energy homeostasis. This condition remains a complex phenomenon that involves several genetic defects and environmental stresses, such as obesity. A complete understanding is required to understand the entire itinerary and functional consequences of insulin resistance to develop a potent drug in diabetes management [1]. Furthermore, drug monotherapy is challenging to provide the desired effect in controlling blood glucose levels in type 2 diabetes patients. Combination therapy which has different mechanisms, is needed for maintaining the requirement of therapeutic management. However, this strategy meets some disadvantages regarding the increasing of drug side effects, toxicity, and interactions. Another alternative is the search for a drug molecule that selectively modulates different targets and improving the balance of efficacy and safety compared to single target agents [2].

This approach aligns with the paradigm that has begun to change in natural product drug discovery, the "herbal shotgun", which utilizes the synergy of multi constituents with varied targets [3]. Research on antidiabetic drugs from Tinospora crispa (T. crispa) are still using the "silver bullet" approach, which is oriented towards the single active principle with one target. As a result, the information obtained was not comprehensively explaining the activity of T. crispa, yet it was closer to the activity of specific metabolites contained in T. crispa [4–12]. Research on medicinal plants conducted by Wink et al. also supports the herbal shotgun approach. It was reported that the combination of metabolite compounds in the medicinal plant had a better effect than the single isolate of metabolite compound [13]. This fact requires more recent research using the herbal shotgun approach so that the activity of medicinal plants can be comprehensively reveal [3].

**Fig 1:**
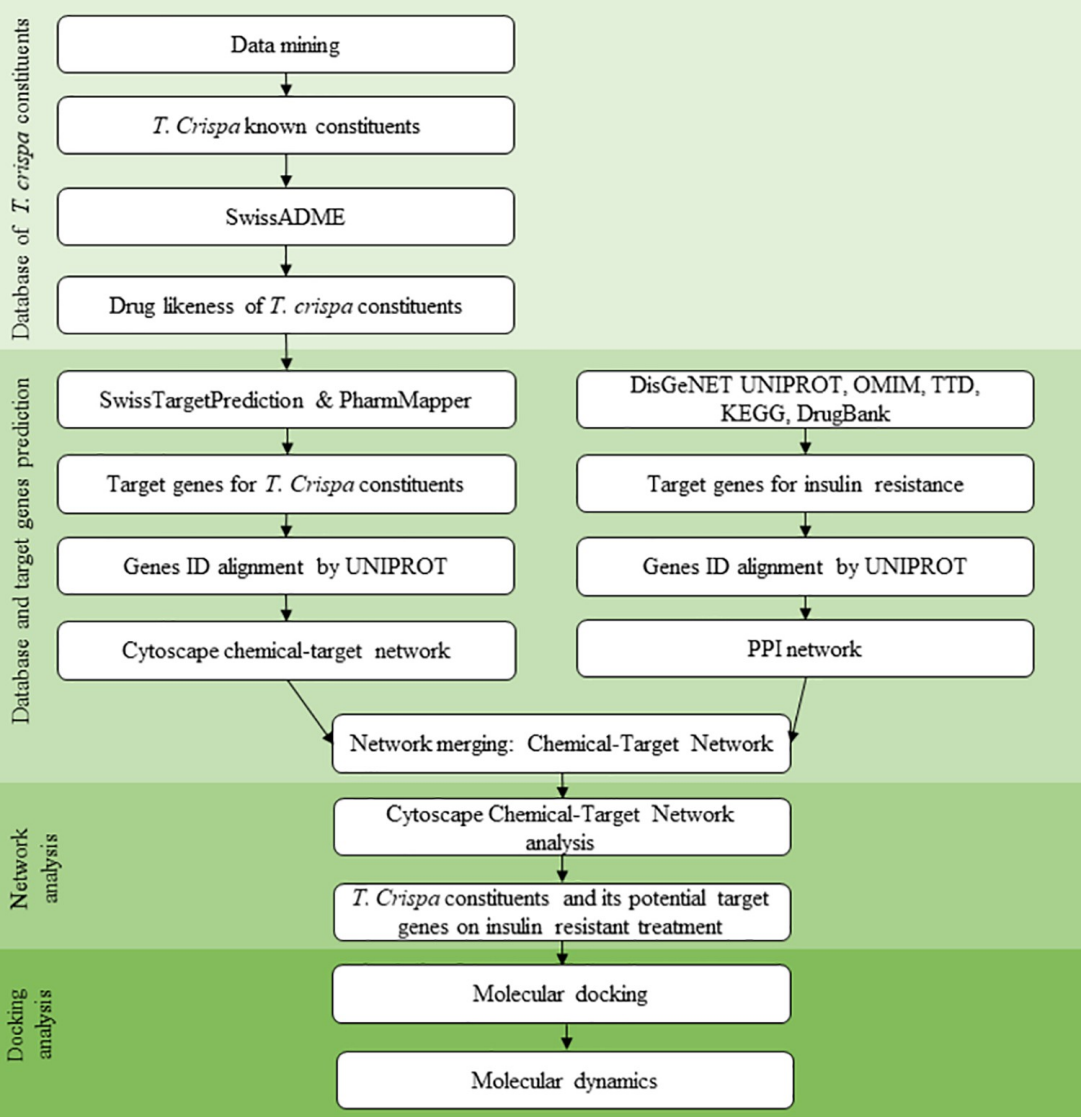
General workflow of present work using network pharmacology and molecular docking analysis.

*T*. *crispa* as a multi-constituent preparation has not known its action mechanism in insulin sensitization activity. It needs to be explored more extensively using a comprehensive approach *in silico* prediction such as network pharmacology analysis. The current development of bioinformatics technology allows researchers to simultaneously predict the action mechanism of medicinal plant constituents as a multicomponent therapeutic activity. This study aims to find a significant therapeutic target in the pathogenesis of insulin resistance targeted by the active compound of T. crispa. The known therapeutic target of compounds contained in T. crispa was analyzed using network pharmacology to determine its potential activity as an insulin sensitizer. The most significant protein targets then verified its binding with T. crispa constituents by molecular docking and molecular dynamics to verify its interactions (Fig 1).

## Material and methods

### Constructing database of known *Tinospora crispa* chemical constituents

Data of chemical constituents in *T*. *crispa* was acquired from former research related to *T*. *crispa* constituent identification [4–12]. Database constructing the 2D structure was made in sdf format were collected from PubChem, ChemSpider (http://www.chemspider.com/), or drawn using MarvinSketch (https://chemaxon.com/products/marvin) [14]. Each 2D structure then changed to a 3D structure using MarvinSketch and saved as PDB format [15]. The 2D structure was needed for druggability analysis in each component, besides the 3D structure for molecular docking analysis. Its compound’s drugability was predicted by the SwissADME database (http://www.swissadme.ch/), including its drug-likeness based on Lipinsky rules of five and its oral bioavailability prediction [16].

### Constructing a database of protein target involved in insulin resistance

Target proteins related to insulin resistance were retrieved from DisGeNET, UniProt, OMIM, TTD, KEGG, and DrugBank database [17–21]. Data retrieval from DisGeNET were done with the keyword "Insulin resistance" (CUI: C0021655) in the diseases search engine column. The retrieved data was a summary of gene-disease associations that have been downloaded in xlsx format (80 genes). Data retrieval from UniProt was done using the human disease option in supporting data and input keyword "Diabetes mellitus, non-insulin-dependent" (DI-02060). Data were downloaded in tab file format (23 genes). Data from both data sources were merging in Cytoscape 3.7.2 software (https://cytoscape.org/) using UniProt ID as their identity to eliminate double listed proteins [18, 22].

### Constructing target proteins of *Tinospora crispa* phytochemical constituents

The simplified molecular-input line-entry specification (SMILES) information of 56 TC constituents was submitted in SWISS Target Prediction (http://www.swisstargetprediction.ch/) PharmMapper to obtain its target prediction. Only human (*Homo sapiens*) target protein was set in the data retrieval [23, 24]. Protein ID was aligned using UniProt ID to synchronize protein ID and eliminate double listed protein [18]. The aligned target proteins ID were arranged in excel format to be imported to Cytoscape 3.7.2, be visualized, and analyzed its degree of connectivity [22].

### Prediction of significance target protein related insulin resistant

Protein-protein interaction (PPI) was generated by submitting all UniProt IDs of the proteins constructed from the previous step into the STRING database (https://string-db.org/) using multiple proteins option [25]. *Homo sapiens* was set as the only organism, and the interaction score confidence was set at the highest confidence (0.900). PPI data is then downloaded in tsv format. Furthermore, the PPI network was analyzed using Cytoscape 3.7.2 software to discover the rank of significance protein based on the degree of connectivity score [22].

### Prediction of *Tinospora crispa* phytoconstituents target genes and their intersection on insulin resistant related target

All the phytochemical *T*. *crispa* compounds were predicted its target by SwissTargetPrediction and PharmMapper [23, 24]. To comprehensively understand its molecular mechanism, all compound-target data were visualized by Cytoscape 3.7.2 to reveal the interaction network between compound-target. Furthermore, the network then merged with the PPI network to discover a new network, a compound-target network of *T*. *crispa* targeted to insulin resistance.

### Ligand and protein preparation for in silico molecular docking

Molecular docking was conducted towards the significant proteins in insulin resistance pathogenesis and was targeted by constituents of *T*. *crispa* at once. Target proteins that meet the criteria were PIK3R1, PTPN1, PPARG, INSR, EGFR, TNF, and AKT2. The 3D structure of *T*. *crispa* constituents act as ligands (file type.pdb) were opened using AutoDock Tools 1.5.6. software (Molecular Graphics Laboratory, The Scripps Research Institute) [15]. Water molecules were removed, and chain contained active site was chosen. The chosen structure is then extracted from its native ligand. The 3D crystal structures of target proteins were selected from Protein Data Bank (PDB) (http://www.rcsb.org/pdb/) [26]. Protein structures with PDB accession number 3S2A were used for PIK3R1 target, 1LQF for PTPN1 target, 6O67 for PPARG target, 5E1S for INSR, 6D8E for EGFR target, 5MU8 for TNF target, and 5D0E for AKT2 target. All protein models were prepared by the addition of polar hydrogen and the addition of Gasteiger charges. Ligands were prepared with a torsion value less than 32. Both ligands and protein models were saved as a pdbqt file. Preparation steps were done using AutoDock Tools 1.5.6 software.

### Molecular docking

Molecular docking was generated using AutoDock Vina (Molecular Graphics Laboratory, The Scripps Research Institute) for initial screening of all *T*. *crispa* constituents followed by AutoDock Tools 1.5.6 only for the top 3 constituents with the lowest binding energy [15, 27]. The ligands were set as rigid structures with an active site was set visually at the centre of the cavity docked by native ligand from each protein model. The grid box was adjusted at 40 x 40 x 40 Angstrom in x, y, and z-axis, respectively. Molecular docking was run in the Lamarckian Genetic Algorithm after validation. Redocking the native ligand with its protein in specific parameters with an RMSD value less than 2 Amstrong remains a valid docking parameter [28]. The results were shown in the value of its lowest binding energy (kcal/mol) calculated by total intermolecular energies, including hydrogen bonds energy, Van der Walls energy, desolvation energy, and electrostatic energy. The more negative energy was, the better ligand-protein binding [29]. The result of ligan-protein complexes was visualized using PyMOL (https://pymol.org/2/). The docking results were analyzed in LigPlot+ to evaluate the accurate binding interaction between ligands and proteins [30].

### Molecular dynamics simulation

Molecular dynamics simulation was carried out using GROMACS (Groningen Machine for Chemical Simulation) software package [31]. Previously docked complex ligand-protein was subjected to 24 ns dynamic simulations in explicit water model using CHARMM36 for the protein and CGenFF force field for the ligand [32, 33]. TIP3P model was used to represent solvation on water molecules using a periodic cubic box with 1.0 nm minimum distance from any atom in the protein to the walls of the cubic box [34]. The whole system was neutralized to pH 7 by adding counter ions, followed by energy minimization, heating and equilibration by running 100 ps of NVT (isothermal-isochoric) and NPT (isothermal-isobaric) ensemble. The production step was carried out at 300K and around 1 atmospheric pressure for 24 ns with a time step of 2 fs. Hydrogen bonds, root mean square deviation (RMSD) of protein (backbone and side chains), as well as root mean square fluctuation (RMSF) of amino acid residues were used to evaluate the ligand-protein interaction. The RMSD and RMSF were plotted using GRACE v5.1.25.

## Results

### Database of *Tinospora crispa* phytoconstituents

A total of 56 compounds were found in the literature related to *T*.*crispa*. 2D and 3D structures of all compounds were collected from PubChem, ChemSpider, or drawn using Marvin Sketch [14]. Its compounds druggability were predicted using SwissADME, including its drug-likeness based on Lipinsky rules of five and its oral bioavailability prediction (Table 1) [16]. The molecular weight range from 135.13 to 714.71, with Adenine as the lightest molecule and Borapetoside H as the heaviest molecule. Predicted gastrointestinal absorption showed 34 of 56 compounds classified as highly absorbed in the GI tract. Drug likeness was assessed through the Lipinski Rules of Five, with 37 compounds meet the five criteria required by Lipinski, while 19 other compounds have violations of Lipinski’s rules of 1–3 violations.

**Table 1:** Prediction of *Tinospora crispa* know phytoconstituents and its druggability [16].

Molecule	Formula	MW (Dalton)	GI abs	Lipinski
(-)-Secoisolariciresinol	C20H26O6	362.42	High	0
N-Acetylnornuciferine	C20H21NO3	323.39	High	0
N-cis-Feruloyltyramine	C18H19NO4	313.35	High	0
N-Formylanonaine	C18H15NO3	293.32	High	0
N-trans-Feruloyltyramine	C18H19NO4	313.35	High	0
Tembetarine	C20H26NO4	344.42	High	0
Tinoscorside A	C30H37NO13	619.61	Low	3
(-)-Litcubinine	C18H20NO4	314.36	High	0
Borapetol A	C20H24O7	376.4	High	0
Borapetol B	C21H26O7	390.43	High	0
Borapetoside A	C26H34O12	538.54	Low	2
Borapetoside B	C27H36O12	552.57	Low	2
Borapetoside C	C27H36O11	536.57	Low	2
Borapetoside D	C33H46O16	698.71	Low	3
Borapetoside E	C27H36O11	536.57	Low	2
Borapetoside F	C27H34O11	534.55	Low	2
Cycloeucalenone	C30H48O	424.7	Low	1
Dihydrodiscretamin	C19H18NO4	324.35	High	0
Luteolin 4’-methyl ether 7-glucoside	C22H22O11	462.4	Low	2
Makisterone C	C29H48O7	508.69	Low	2
N-acetylanonaine	C19H17NO3	307.34	High	0
N-acetylanornuciferine	C20H21NO3	323.39	High	0
N-formylanonaine	C18H15NO3	293.32	High	0
N-formylasimilobine 2-O-β-D-glucopyranoside	C24H29NO7	443.49	High	0
N-trans-caffeoyltyramine	C17H17NO4	299.32	High	0
Paprazine	C17H17NO3	283.32	High	0
Rumphioside A	C27H36O13	568.57	Low	2
Rumphioside B	C28H38O13	582.59	Low	2
(-)-Litcubinine	C18H29NO4	340.56	High	0
Berberine	C20H29NO4	347.45	High	0
Beta sitosterol	C29H50O	414.71	Low	1
Uridine	C9H12N2O6	244.2	Low	0
Tyramine	C8H11NO	137.18	High	0
Tinocrispol A	C21H24O7	388.41	High	0
Syringin	C17H24O9	372.37	Low	0
Syringaresinol	C22H26O8	418.44	High	0
Stigmasterol	C29H48O	412.69	Low	1
Secoisolariciresinol	C20H26O6	362.42	High	0
Salsolinol	C10H13NO2	179.22	High	0
Palmatine	C21H22NO4	352.4	High	0
Rumphiol E	C20H24O6	360.4	High	0
N-demethyl-N-formyldehydronornuciferine	C19H17NO3	307.34	High	0
Magnoflorine	C20H24NO4	342.41	High	0
Lysicamine	C18H13NO3	291.3	High	0
Luteolin 4’-methyl ether 7-glucoside	C22H22O11	462.4	Low	2
Luteolin 4’-methyl ether 3’-glucoside	C22H22O11	462.4	Low	2
Jatrorrhizine	C20H20NO4	338.38	High	0
Higenamine	C16H17NO3	271.31	High	0
Genkwanin	C16H12O5	284.26	High	0
Cycloeucalenol	C30H50O	426.72	Low	1
Columbamine	C20H20NO4	338.38	High	0
Borapetoside H	C33H46O17	714.71	Low	3
Borapetoside G	C26H32O12	536.53	Low	2
Apigenin	C15H10O5	270.24	High	0
Adenosine	C10H13N5O4	267.24	Low	0
Adenine	C5H5N5	135.13	High	0

SWISS target prediction and DisGeNET identified that 56 constituents of *T*. *crispa* were documented to have 5666 protein-specific targets [17, 23]. Those 5666 target proteins were then analyzed by Cytoscape 3.7.2 to find out its intersection with insulin-resistant target protein [22].

### Protein-protein interaction networks related to insulin resistance

**Fig 2:**
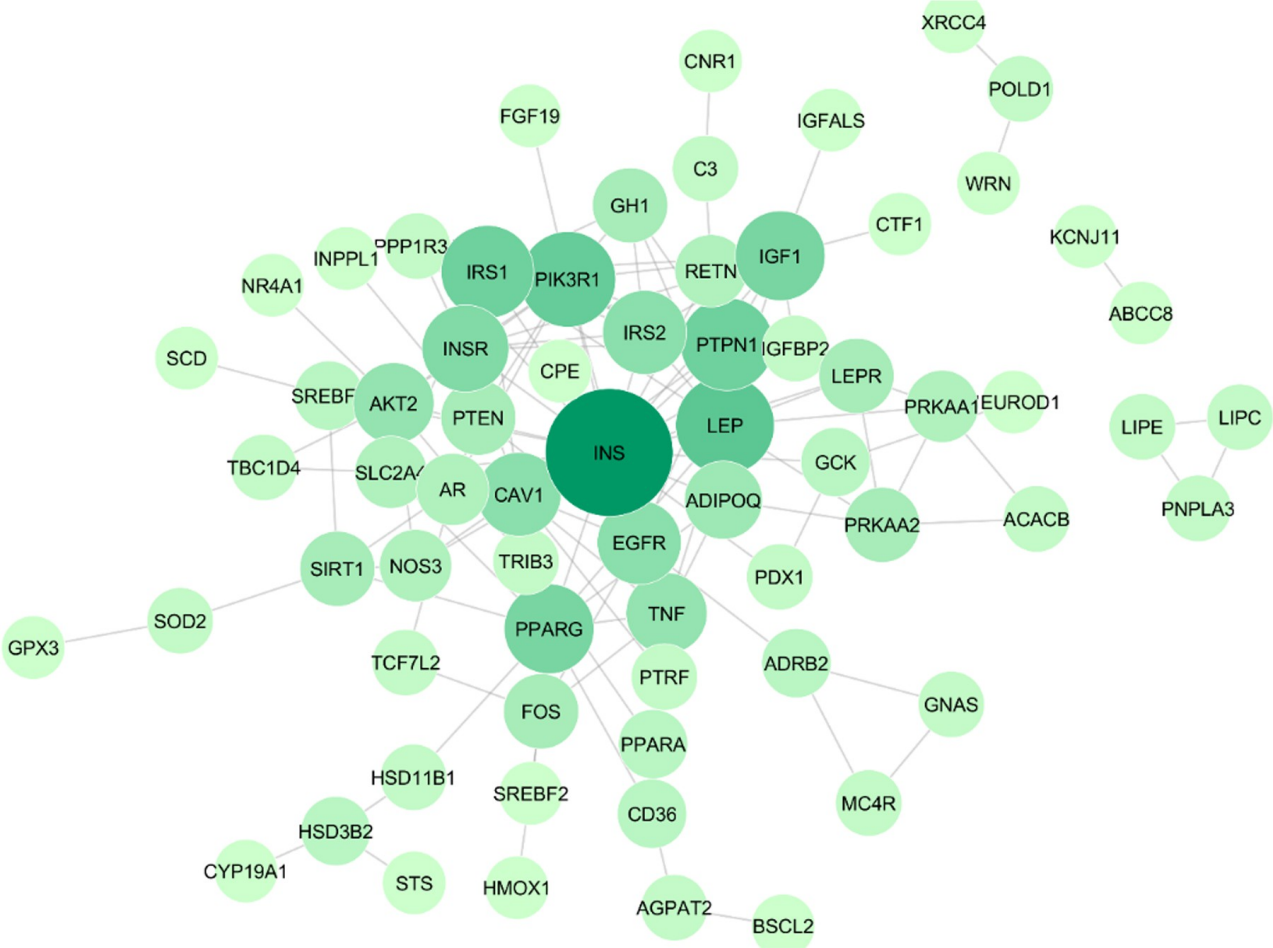
PPI network of protein related insulin resistance in *Homo sapiens*. The darker purple color and the bigger its nodes indicate higher degree of protein in the network.

267 targets were documented to have relation to insulin resistance pathogenesis. All targets were identified its interaction using STRING database [25]. Those interactions construct a PPI network which is then assessed by Cytoscape 3.7.2 software for the degree of connectivity value. The analysis results were displayed in Fig 2 in white to green gradation. The higher the degree of a node (gene) in the network is marked with a darker green colour. The higher the degree value indicates that the protein has a more significant role in the pathogenesis of insulin resistance. The top degree ranks were INS, LEP, PI3K, PTPN1, IRS1, PPARG, IGF1, INSR, CAV1, EGFR, IRS2, TNF, and AKT2, respectively. The degree rating of the entire PPI network is shown in Fig 2. The exact degree value was shown in S1 Table.

### Interaction of lead compounds of *Tinospora crispa* on insulin resistant related target proteins

**Fig 3:**
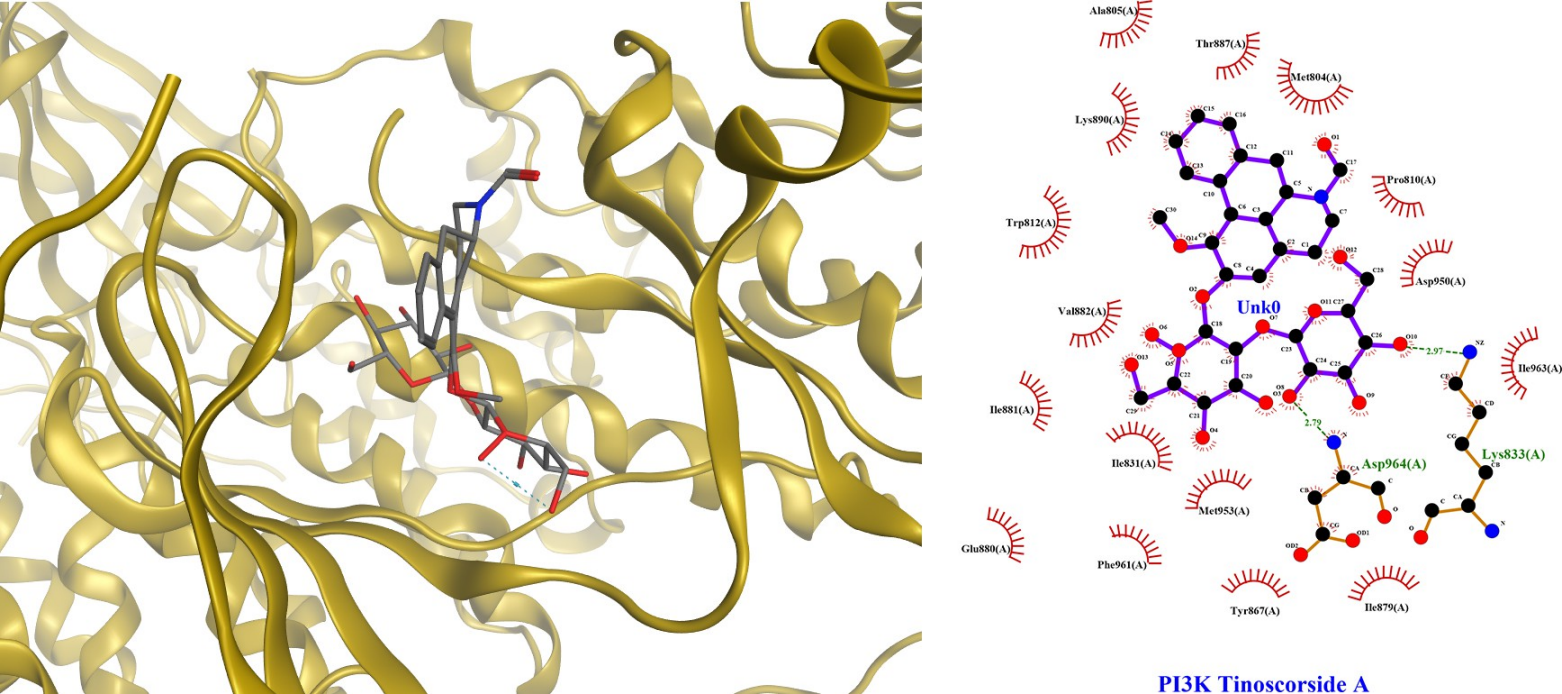
Docking sketch map of Tinoscorside A interaction with the crystal structure of PI3K. (A) Pocket view of Tinoscorside A binding with PI3K active site. (B) 2D docking pattern of Tinoscorside A with amino acids hydrogen bonds LYS833 and ASP964.

**Fig 4:**
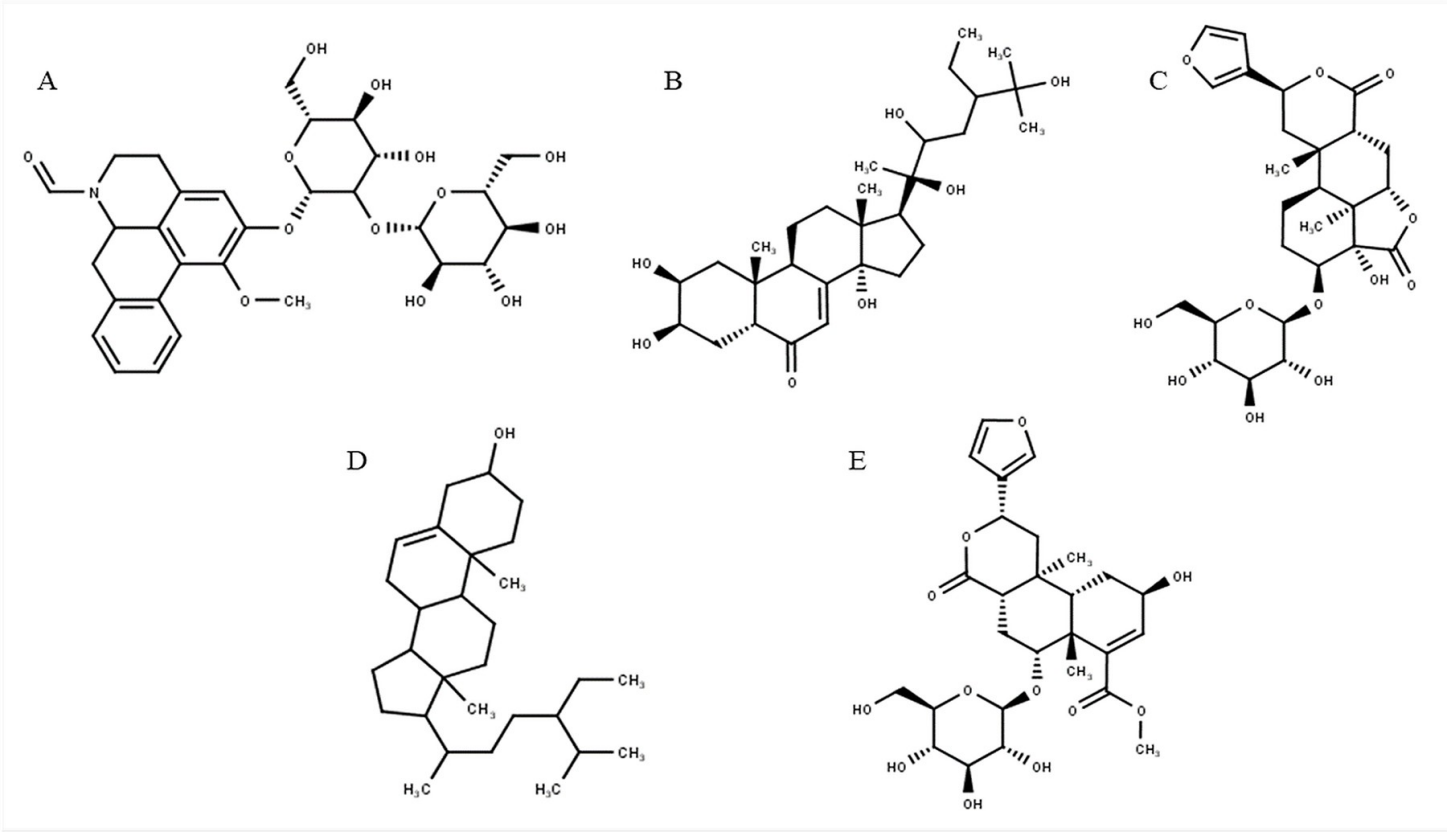
Structure of active constituents prediction of *T*. *crispa* as insulin sensitizer. (A) Tinoscorside A (B) Makisterone C (C) Borapetoside A (D) β Sitosterol (E) Borapetoside B. All structures were drawn using MarvinSketch based on the previous studies [6].

We conducted the molecular docking strategy to prove the underlying interactions between *T*. *crispa* predicted active phytoconstituents to insulin resistance. Molecular docking was conducted on seven target proteins that meet the criteria of high connectivity value, targeted by *T*. *crispa* constituents and was involved in insulin resistance pathogenesis. Those targets were PIK3R, PTPN1, PPARG, INSR, EGFR, TNF, and AKT2. The best docking interaction was PI3K with Tinoscorside A as a ligand (binding energy -11.64), as shown in Table 2. Its binding sketch map was shown in Fig 3 as well. The detailed docking scores of *T*. *crispa* targeted to 7 significance proteins were shown in S3 Table, and the docking sketch maps were shown in S1 Fig. The structures of the main constituents of *T*. *crispa* were shown in Fig 4.

**Table 2:** Docking scores of the lowest ligand-protein binding energy of *T*. *crispa* constituents and significant proteins in insulin resistance.

Target protein	Compound	Ki (nM)	The lowest binding energy (kcal/mol)
PI3K	Tinoscorside A	2.94	-11.64
INSR	Tinoscorside A	4.19	-11.43
AKT2	Makisterone C	5.17	-11.3
PPARG	Borapetoside A	51.96	-9.94
EGFR	Tinoscorside A	191.99	-9.16
PTPN1	Beta sitosterol	453.59	-8.65
TNF	Borapetoside B	1.62x10^3^	-7.90

### Molecular dynamics simulation

**Fig 5:**
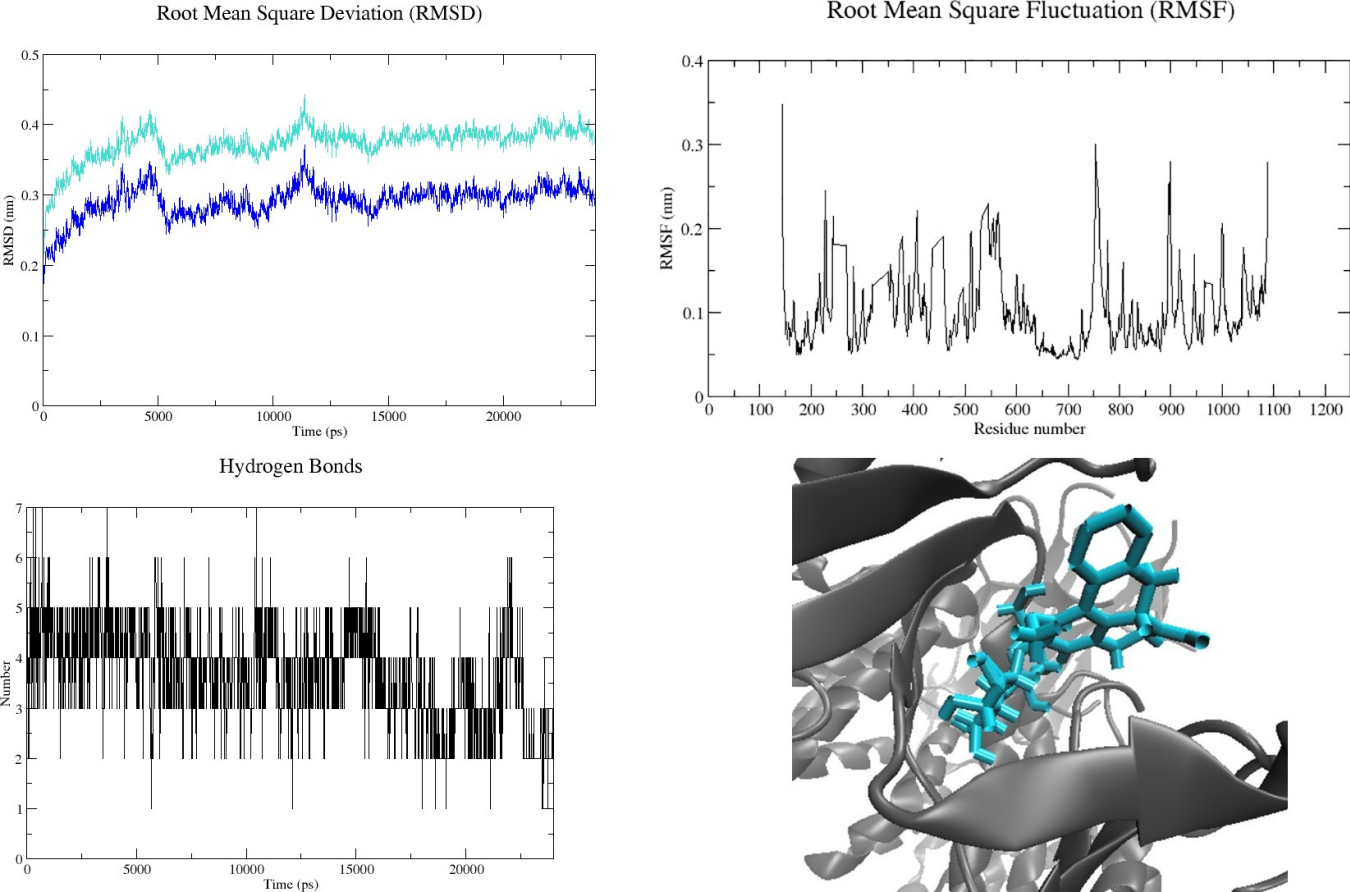
Molecular dynamics (MD) simulation analysis of Tinoscorside A with PI3K. **(A) root-mean square deviation (RMSD) of the complex against the initial crystal structure.** The The blue graph represent RMSD of C alpha and the turquoise one represent RMSD of the side-chain; **(B) root-mean square fluctuation (RMSF) of the C alpha residue; (C) The dynamics of hydrogen bond number along MD simulation; (D) the representative of the Tinoscorside A-PI3K complex after 24 ns MD simulation**.

To clarify the protein-ligand stability and protein structural flexibility between the docked complex of PI3K and Tinoscorside, we performed a 24 ns MD simulation using GROMACS software. Protein and ligand root-mean-square deviation (RMSD) information of C alpha and side chains of the complex are presented in Fig 5A. The root-mean-square fluctuation (RMSF) of amino acid residues are presented in Fig 5B. RMSD calculation shows that the complex has nearly constant RMSD since 15 ns MD production. The maximum RMSF of the C alpha atom is 2.5–3.0 nm, except the initial C alpha residue, which reaches 3.5 nm. Hydrogen bonds between protein and ligand were ranged from 1–7 bonds in each frame (Fig 5C). There were 20 hydrogen bonds formed between protein-ligand during the MD simulation. The amino acid residues involved in forming hydrogen bonds are Ala805, Ser806, Lys833, Asp841, Tyr867, Glu880, Val882, Thr887, Asp964, and Lys890. The representation of the complex after 24 ns MD simulation was performed in Fig 5D.

### Compound-target network

**Fig 6:**
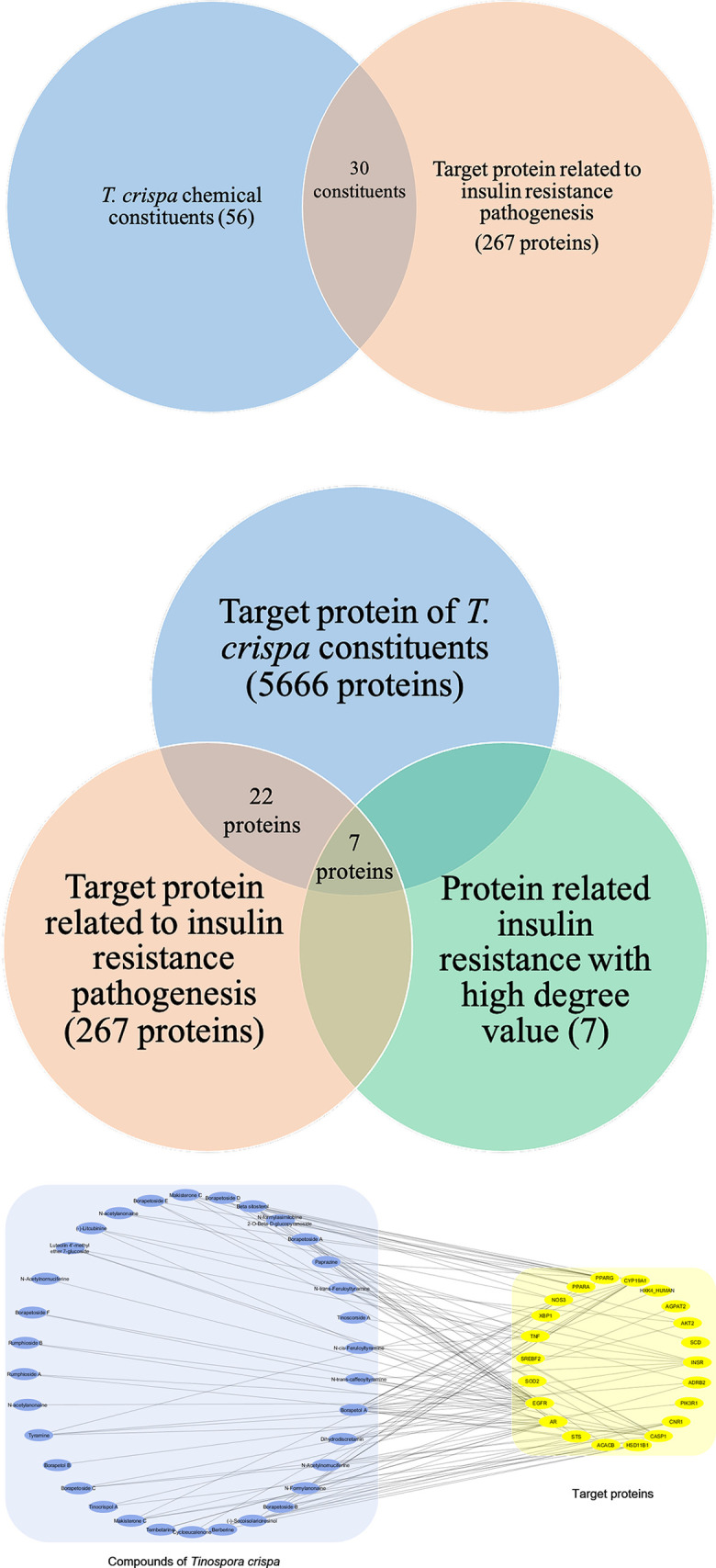
**(A)** Intersection Venn diagram of *T*. *crispa* constituents and target protein related insulin resistance. There were 30 constituents of *T*. *crispa* that were predicted to be targeted to insulin resistance target protein. **(B)** Intersection Venn diagram between predicted target protein of *T*. *crispa* constituents, target proteins of insulin resistance pathogenesis, and high degree of connection value of target proteins of insulin resistance. There were 7 target proteins that meet the three intersections. In addition there were 22 proteins targeted by *T*. *crispa* constituents that were shown visually in Fig 6C. **(C)** Compound-target network of *T*. *crispa* constituents targeted to insulin resistance pathogenesis. The blue dots represent *T*. *crispa* constituents, the yellow dots represent protein targets, and the connecting lines represent compound-target interaction.

To further discover the potential target and therapeutic pathway of *T*. *crispa* constituents in ameliorating insulin resistance, the targets of insulin resistance were merged with the target of *T*. *crispa* (Fig 6A). The compound-target network of *T*. *crispa* against insulin resistance was shown in Fig 6C. After the network merging using Cytoscape 3.7.2, 30 *T*. *crispa* constituents were reserved as the main active constituents in *T*. *crispa* treating insulin resistance. In addition, 22 targets were reserved as the main targets of TC in treating insulin resistance. Moreover, 7 targets were revealed to have significant protein in insulin resistance pathogenesis and also targeted by *T*. *crispa* constituents (Fig 6B). The complete *T*. *crispa* constituents and their targets were shown in S2 Table.

### Discussion

The present study tried to collect and investigate clues from the known phytoconstituents of *T*. *crispa* in ameliorating insulin resistance. Investigations were conducted using in silico approaches, including network pharmacology, molecular docking, and molecular dynamics simulation. We elucidated the potential therapeutic network of insulin resistance and intersected this network with the *T*. *crispa* phytoconstituents potential target network. Both of the networks were complicated networks with 5666 and 267 nodes. The intersection showed us only the protein-related insulin resistance targeted by *T*. *crispa* constituents. From 267 targets, there were only 22 target proteins that were targeted by *T*. *crispa* constituents.

**Fig 7:**
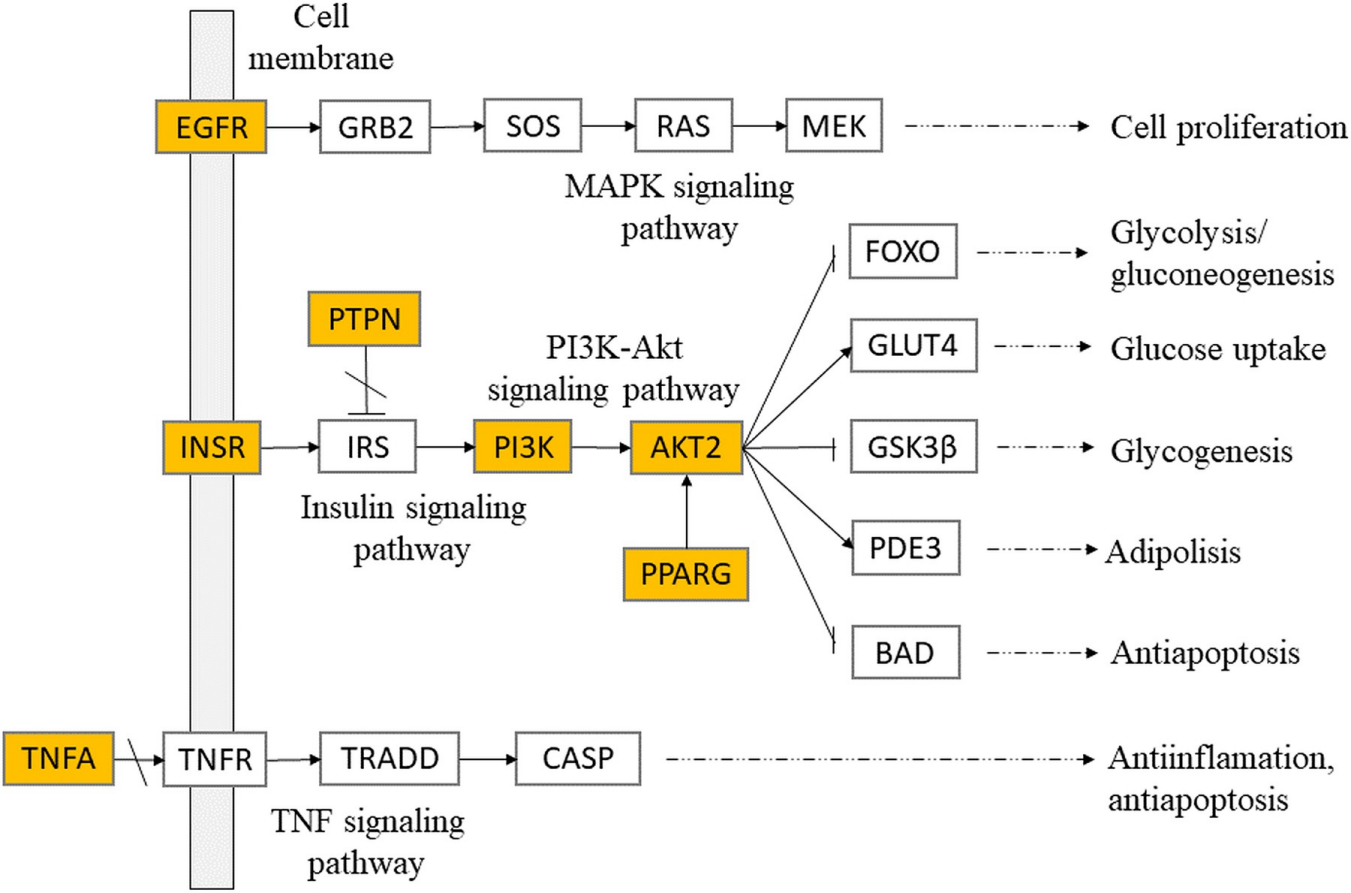
Systematic prediction of 7 significant protein targets and therapeutic pathways of *T*. *crispa* constituents on insulin resistant. The highlighted nodes represent the significant targets of *T*. *crispa* and other nodes represent their related target on insulin resistant. All the presented pathways were summarized by KEGG pathway database [21].

KEGG pathways analysis had indicated several signalling pathways, including insulin signalling, PI3K-Akt signalling, MAPK signalling, and TNF signalling pathway, that play vital roles in the therapeutic mechanism of *T*. *crispa* as insulin sensitizer. Those signalling pathways had close relative to glucose-lowering activity through the regulation of glucose homeostasis, adipolysis, cell proliferation, and antiapoptosis. This showed that *T*. *crispa*, with its various secondary metabolites, had acted as an insulin sensitizer through different pathways (Fig 7). This finding supports Wink *et al*. statement that the activity of a medicinal plant was due to synergistic interactions of several phytoconstituents [13]. The network pharmacology approach provides an insight into prediction in the mode of action of a medicinal plant comprehensively with multiple target therapies [35]. Network pharmacology analysis also informed that the main phytoconstituents of *T*. *cripa* as insulin sensitizer were Tinoscorside A, Makisterone C, Borapetoside A, Beta-sitosterol, and Borapetoside B.

Based on former studies, Borapetoside A-C, and E may be the key of *T*. *crispa* constituents as hypoglycaemic agents [7, 11, 12, 36, 37]. Lam *et al* reported, the isolated Borapetoside A and C had significantly reduced plasma glucose levels but unable to increase insulin secretion in MIN6 pancreatic beta cells [36]. It indicated that they had other mechanisms of hypoglycemic activity. This study predicts the mechanism of action of Borapetoside A through activation of PPARG and Borapetoside B through inhibition of TNF cytokines to bind to their receptor.

Contrary to previous studies that indicated diterpenoids as active constituents, this study presents Tinoscorside A (N-formylasimilobine 2-O-β-D-glucopyranosyl-(1→2)-β- D-glucopyranoside) as a constituent of *T*. *crispa* which has the strongest docking binding with some protein targets including PI3K, INSR, and EGFR respectively. Tinoscorsida A was a member of aporphine alkaloid which has never been proven as part of active compounds of *T*. *crispa* in reducing blood sugar level. This finding indicated that Tinoscorside A could be a potential glucose lowering agent through multiple ways.

The molecular dynamics analysis from the PI3K and Tinoscorside A as the ligand-protein complex with the highest affinity, shows a stable conformation in water solvation under 300 K temperature and 1 atmospheric pressure. It is in line with the docking analysis result. The formation of 20 hydrogen bonds during the MD simulation indicates that the interaction has a high affinity. Two of these bonds are hydrogen bonds reported in molecular docking. Both bonds involve the amino acids Lys833 and Asp964. These two amino acids are active sites that also interact with native ligands in the crystallographic structure of proteins. In addition to these two residues, eight other amino acids are reported to be involved in the formation of hydrogen bonds. Two of them are also included in active sites that interact with native ligands, namely Tyr867 and Val882. In addition, this study also predicted that T. crispa had anti-inflammatory activity through the TNF signalling pathway by inhibiting the binding between TNFA and TNFR.

## Conclusion

The present study highlights the insulin-sensitizing role of *T*. *crispa* on insulin resistance pathogenesis by network pharmacology and molecular docking study. The role of *T*. *crispa* phytoconstituents that served as plant extract with multiple constituents remains still undescribed. Our study provides valuable clues to reveal the prediction of their possible targets and mechanistic pathways of *T*. *crispa*. The present study highlights the insulin-sensitizing role of *T*. *crispa* on insulin resistance pathogenesis by network pharmacology and molecular docking study. The role of *T*. *crispa* phytoconstituents that served as plant extract with multiple constituents remains still undescribed. Our study provides valuable clues to reveal the prediction of their possible targets and mechanistic pathways of *T*. *crispa*. The result was shown that *T*. *crispa* was a promising plant-based drug to be developed as an insulin sensitizer through multiple pathways, including glucose homeostasis, adipolysis, cell proliferation, and antiapoptosis. However, there are several limitations in the current study, including the limitation of *the T*. *crispa* phytoconstituents database that could be more phytoconstituents were not revealed yet and the limitation of the database that serves the activity of the constituents as a single compound neglecting the interaction of the multi-compound itself. Further study on the insulin-sensitizing role of *T*. *crispa* and the underlying mechanism are still urgently to be investigated in other methods such as *in vitro*, *in vivo*, and clinical study evaluation to verify this *in silico* study.

## Supporting information

S1 Fig Docking sketch map of best ligand-binding position of T.crispa phytoconstituents and 7 selected target proteins in insulin resistance. (DOCX)

S1 Table Degree ranking of insulin resistant related targets analyzed by Cytoscape 3.7.2. (DOCX)

S2 Table 30 *T*. *crispa* phytoconstituents and its target proteins [17, 18, 24]. (DOCX)

S3 Table Molecular docking result of *T*. *crispa* phytoconstituents to 7 selected target proteins using AutoDock. (DOCX)
