# Supplementary material for: Network pharmacology integrated molecular dynamics reveals the bioactive compounds and potential targets of Tinospora crispa Linn. as insulin sensitizer
Source: PLoS One. 2022 Jun 23;17(6):e0251837. doi: 10.1371/journal.pone.0251837 (PMC9223613; doi:10.1371/journal.pone.0251837)
Supplement: S1 Fig — (DOCX) [file pone.0251837.s001.docx]

**S1 Fig. Docking sketch map of best ligand-binding position of T.crispa phytoconstituents and 7 selected taget proteins in insulin resistance**.

| a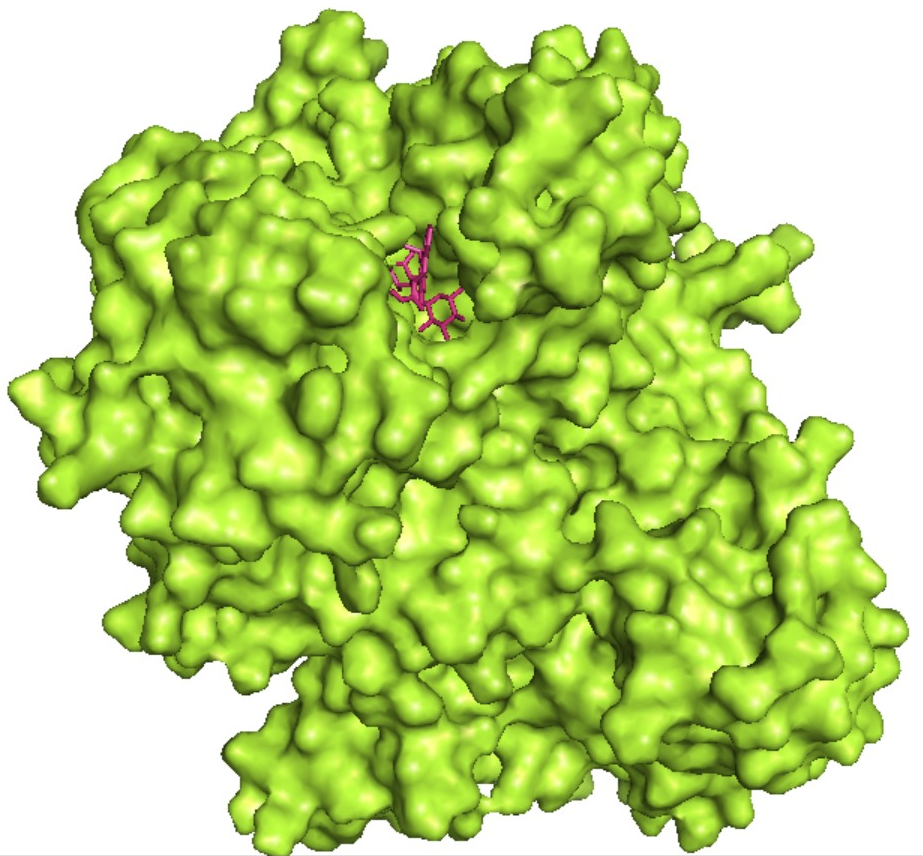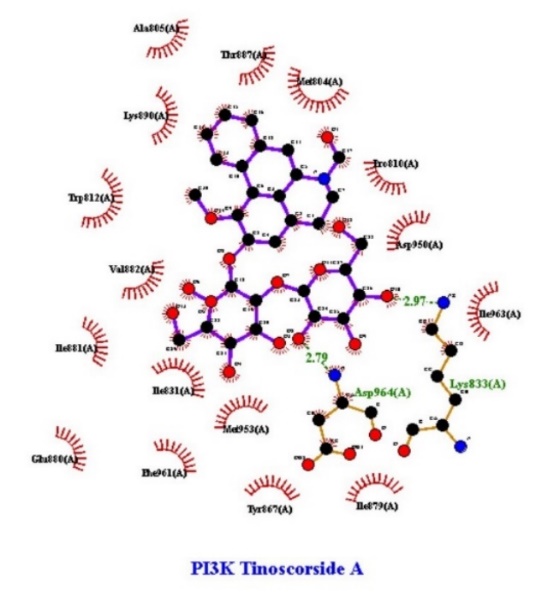 | b 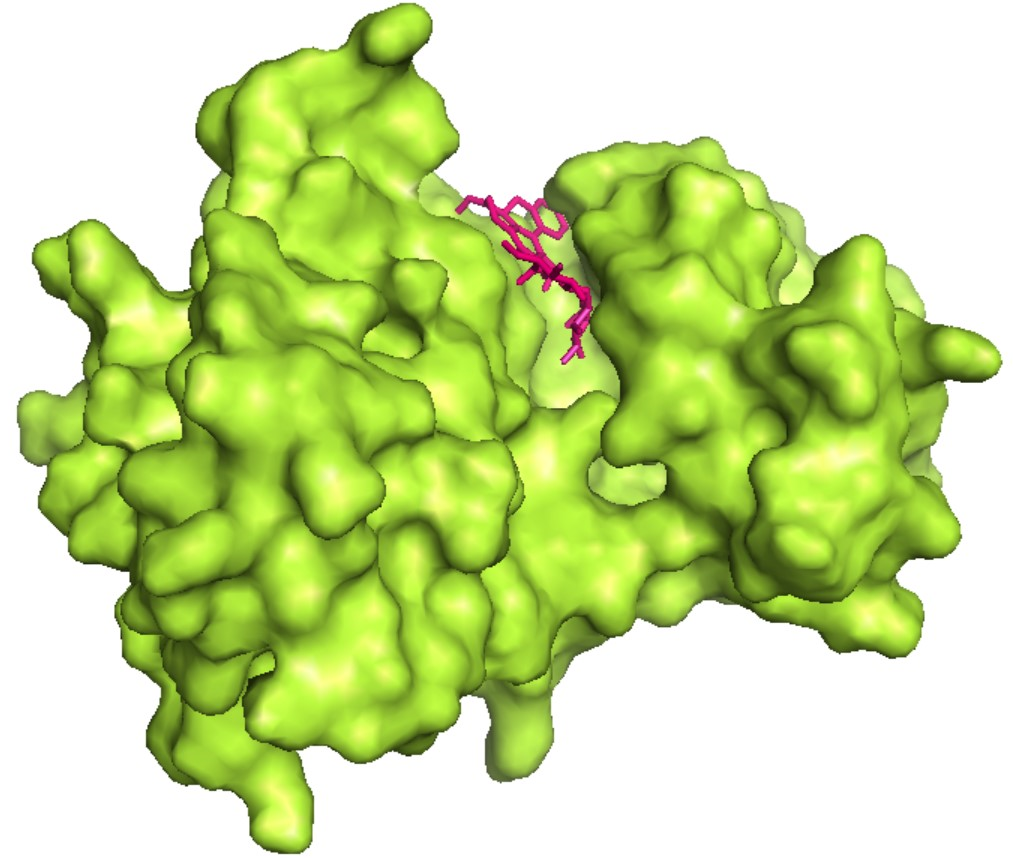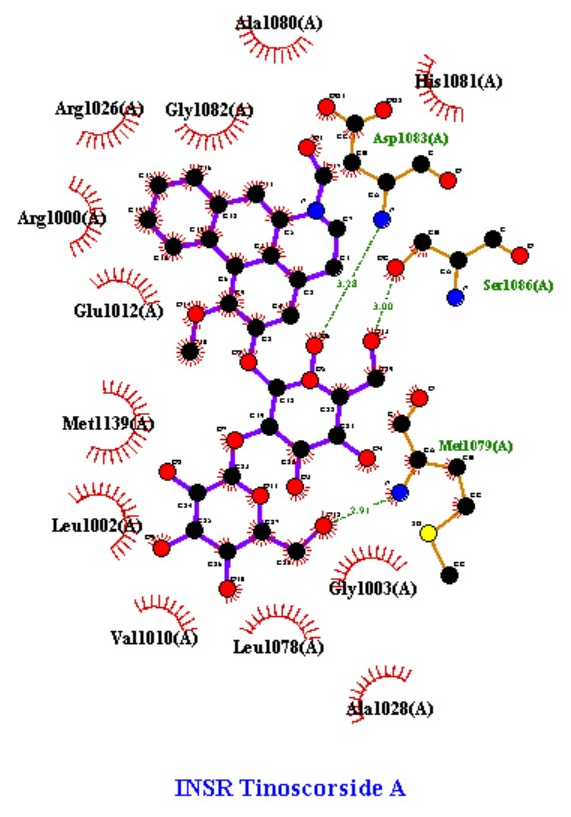 |
| --- | --- |
| c 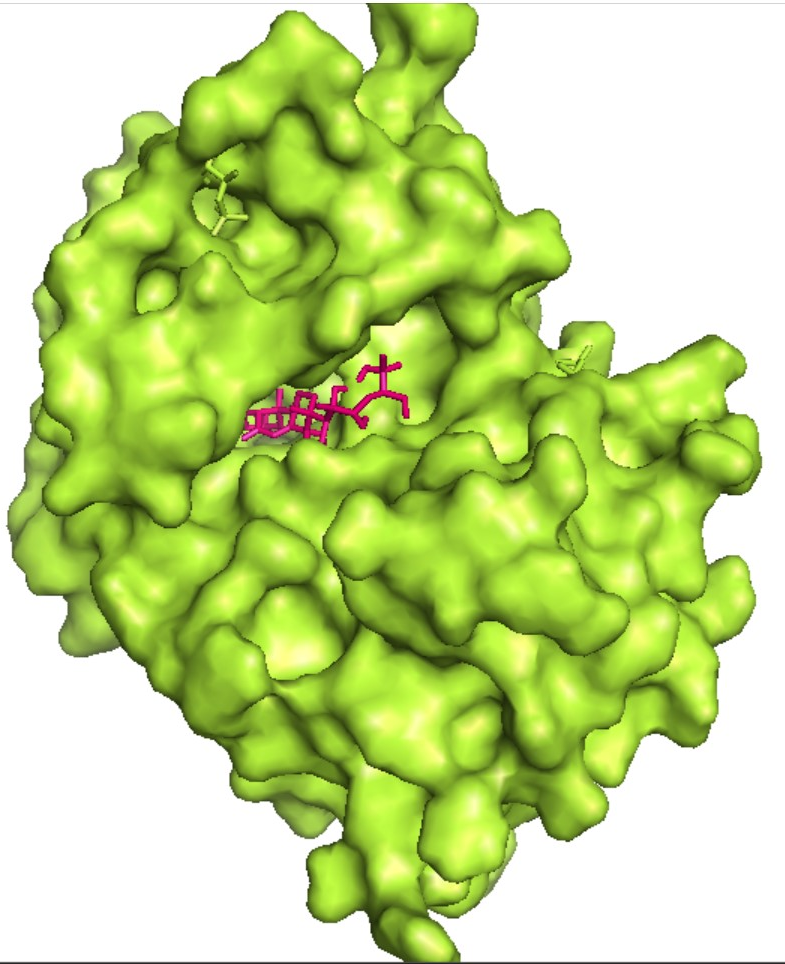 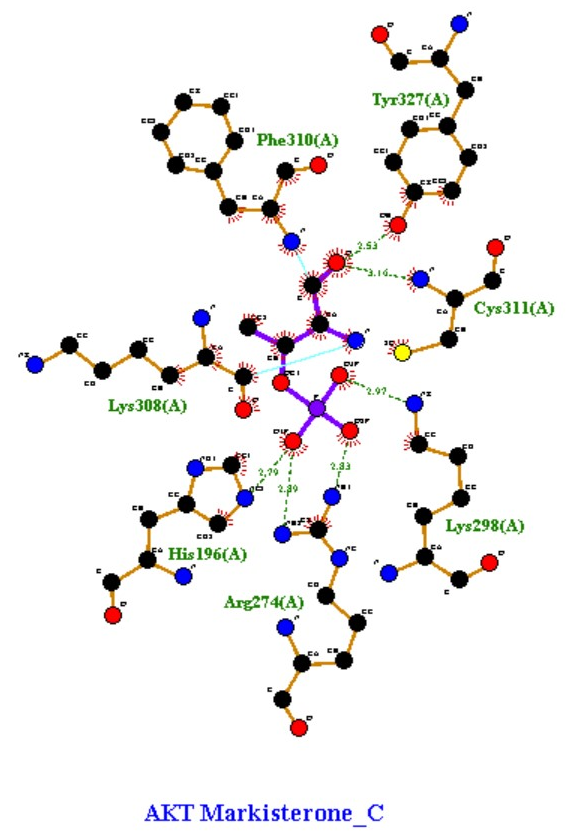 | d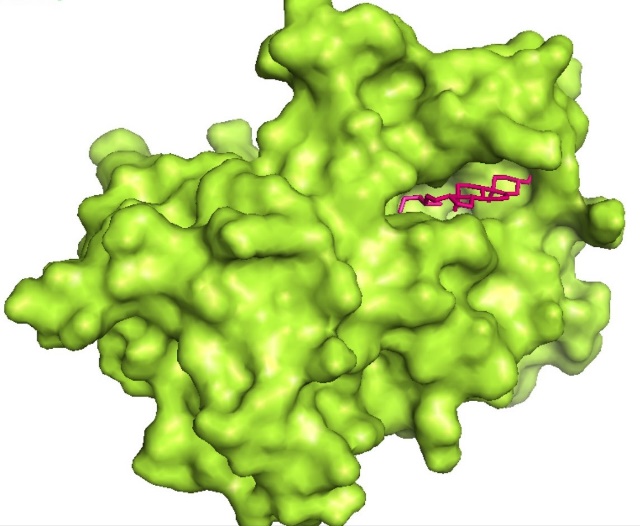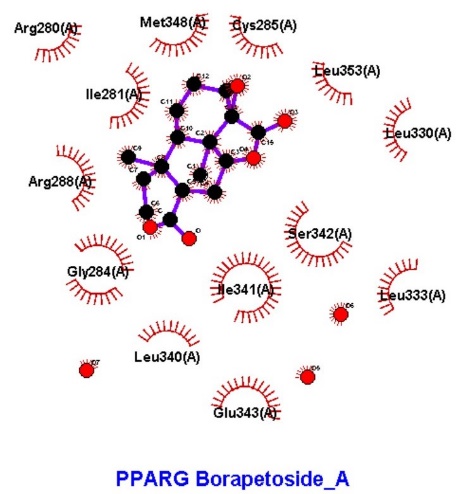 |
| e 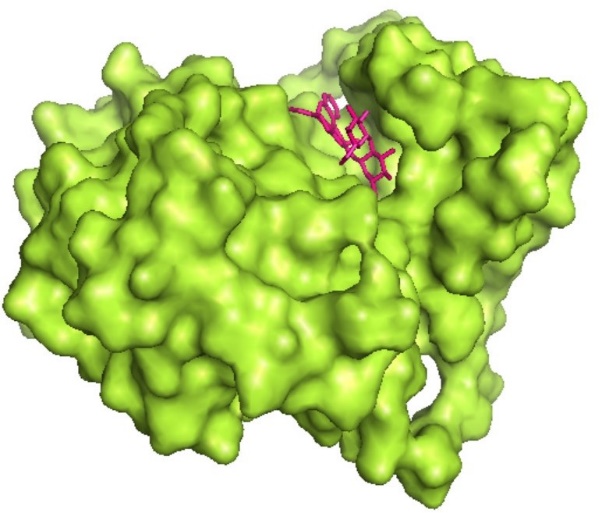 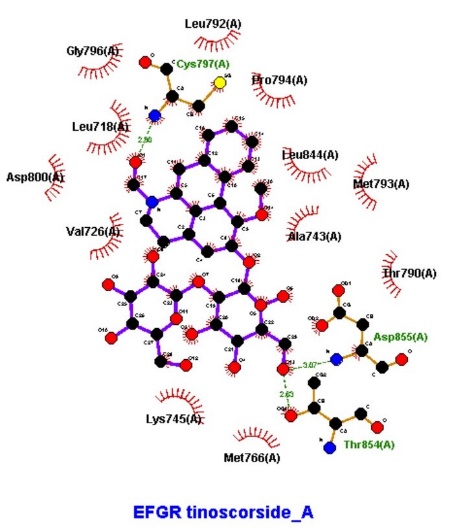 | f 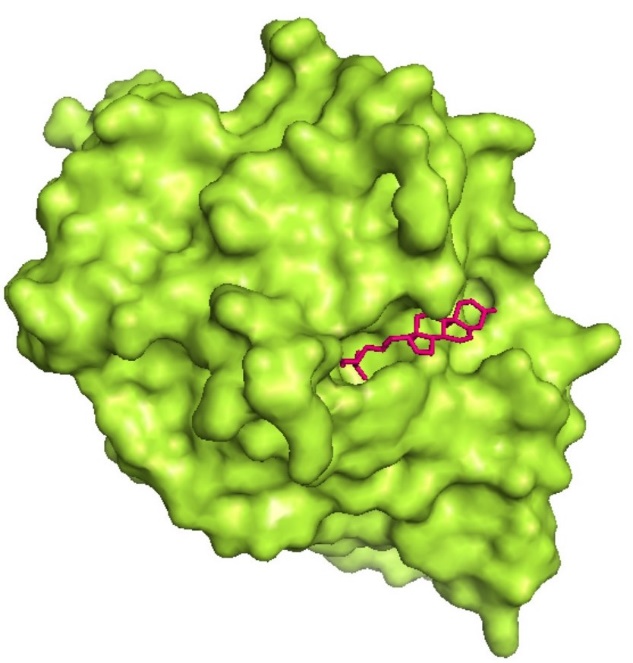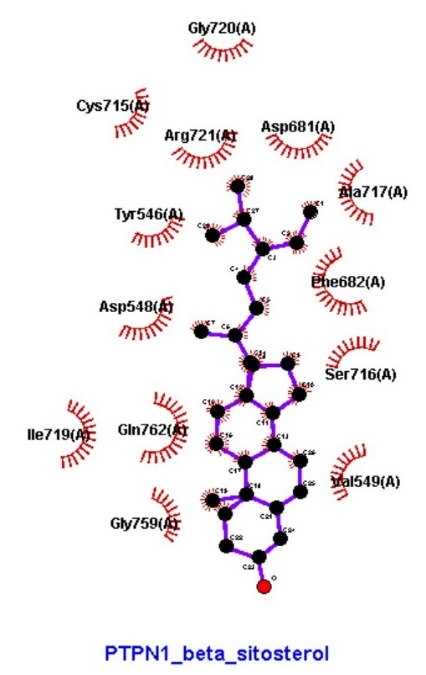 |
| g 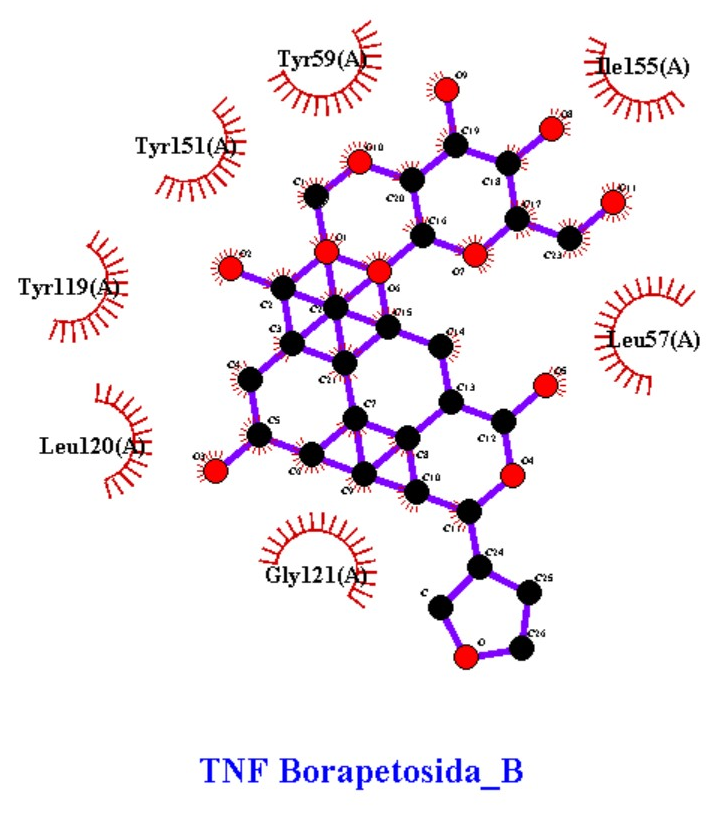 | |
