# Supplementary material for: Network pharmacology integrated molecular dynamics reveals the bioactive compounds and potential targets of Tinospora crispa Linn. as insulin sensitizer
Source: PLoS One. 2022 Jun 23;17(6):e0251837. doi: 10.1371/journal.pone.0251837 (PMC9223613; doi:10.1371/journal.pone.0251837)
Supplement: S1 Table — (DOCX) [file pone.0251837.s002.docx]

**S1 Table. Degree ranking of insulin resistant related targets analysed by Cytoscape 3.7.2**

| **Gene name** | **Uniprot ID** | **Degree** |  | **Gene name** | **Uniprot ID** | **Degree** |
| --- | --- | --- | --- | --- | --- | --- |
| INS | P01308 | 23 |  | MC4R | P32245 | 2 |
| LEP | P41159 | 13 |  | ACACB | O00763 | 2 |
| PIK3R1 | P27986 | 12 |  | HSD11B1 | P28845 | 2 |
| PTPN1 | P18031 | 11 |  | TCF7L2 | Q9NQB0 | 2 |
| IRS1 | P35568 | 11 |  | TRIB3 | Q96RU7 | 2 |
| PPARG | P37231 | 10 |  | LIPC | P11150 | 2 |
| IGF1 | P05019 | 10 |  | POLD1 | P28340 | 2 |
| INSR | P06213 | 9 |  | GNAS | Q5JWF2 | 2 |
| CAV1 | Q03135 | 8 |  | PTRF | Q6NZI2 | 2 |
| EGFR | P00533 | 8 |  | SOD2 | P04179 | 2 |
| IRS2 | Q9Y4H2 | 8 |  | PNPLA3 | Q9NST1 | 2 |
| TNF | P01375 | 7 |  | LIPE | Q05469 | 2 |
| AKT2 | P31751 | 7 |  | PDX1 | P52945 | 2 |
| ADIPOQ | Q15848 | 6 |  | TBC1D4 | O60343 | 2 |
| GH1 | P01241 | 5 |  | IGFBP2 | P18065 | 2 |
| PRKAA2 | P54646 | 5 |  | FGF19 | O95750 | 1 |
| SIRT1 | Q96EB6 | 5 |  | NR4A1 | P22736 | 1 |
| FOS | P01100 | 5 |  | CNR1 | P21554 | 1 |
| PTEN | P60484 | 5 |  | STS | P08842 | 1 |
| LEPR | P48357 | 5 |  | XRCC4 | Q13426 | 1 |
| PRKAA1 | Q13131 | 4 |  | BSCL2 | Q96G97 | 1 |
| RETN | Q9HD89 | 4 |  | CTF1 | Q16619 | 1 |
| AR | P10275 | 4 |  | SCD | O00767 | 1 |
| SLC2A4 | P14672 | 4 |  | HMOX1 | P09601 | 1 |
| NOS3 | P29474 | 4 |  | SREBF2 | Q12772 | 1 |
| SREBF1 | P36956 | 3 |  | INPPL1 | O15357 | 1 |
| PPARA | Q07869 | 3 |  | NEUROD1 | Q13562 | 1 |
| CD36 | P16671 | 3 |  | WRN | Q14191 | 1 |
| ADRB2 | P07550 | 3 |  | CPE | P16870 | 1 |
| GCK | P35557 | 3 |  | CYP19A1 | P11511 | 1 |
| HSD3B2 | P26439 | 3 |  | GPX3 | P22352 | 1 |
| C3 | P01024 | 2 |  | IGFALS | P35858 | 1 |
| AGPAT2 | O15120 | 2 |  | ABCC8 | Q09428 | 1 |
| PPP1R3A | Q16821 | 2 |  | KCNJ11 | Q14654 | 1 |
