# Supplementary material for: Network pharmacology integrated molecular dynamics reveals the bioactive compounds and potential targets of Tinospora crispa Linn. as insulin sensitizer
Source: PLoS One. 2022 Jun 23;17(6):e0251837. doi: 10.1371/journal.pone.0251837 (PMC9223613; doi:10.1371/journal.pone.0251837)
Supplement: S2 Table — (DOCX) [file pone.0251837.s003.docx]

**S2 Table. 30 *T. crispa* phytoconstituents and its target proteins^17,18,24^**

| **No** | **Compound** | **Target** | **UniProt ID** |
| --- | --- | --- | --- |
| 1 | (-)-Litcubinine | X-box-binding protein 1 | P17861 |
|  |  | Steryl-sulfatase | P08842 |
|  |  | Adrenergic receptor beta | P07550 |
| 2 | (-)-Secoisolariciresinol | Cannabinoid receptor 1 | P21554 |
|  |  | Androgen Receptor | P10275 |
|  |  | Steryl-sulfatase | P08842 |
|  |  | Cytochrome P450 19A1 | P11511 |
| 3 | Berberine | 11-beta-hyxdroxysteroid dehydrogenase 1 | P28845 |
| 4 | Beta sitosterol | Peroxisome proliferator-activated receptor gamma | P37231 |
|  |  | Peroxisome proliferator-activated receptor alpha | Q07869 |
|  |  | Sterol regulatory element-binding protein 2 | Q12772 |
|  |  | Androgen Receptor | P10275 |
|  |  | Acetyl-CoA carboxylase 2 | O00763 |
|  |  | 11-beta-hydroxysteroid dehydrogenase 1 | P28845 |
|  |  | Acyl-CoA desaturase | O00767 |
|  |  | Cytochrome P450 19A1 | P11511 |
| 5 | Borapetol A | Insulin receptor | P06213 |
|  |  | Peroxisome proliferator-activated receptor gamma | P37231 |
|  |  | Peroxisome proliferator-activated receptor alpha | Q07869 |
|  |  | Nitric-oxide synthase, endothelial | P29474 |
|  |  | Superoxide dismutase [Mn], mitochondrial | P04179 |
|  |  | Epidermal growth factor receptor erbB1 | P00533 |
|  |  | Androgen Receptor | P10275 |
|  |  | Steryl-sulfatase | P08842 |
|  |  | 11-beta-hydroxysteroid dehydrogenase 1 | P28845 |
|  |  | Caspase-1 | P29466 |
|  |  | Serine/threonine-protein kinase AKT2 | P31751 |
|  |  | Glucokinase | P35557 |
| 6 | Borapetol B | Epidermal growth factor receptor erbB1 | P00533 |
| 7 | Borapetoside A | Peroxisome proliferator-activated receptor gamma | P37231 |
|  |  | Epidermal growth factor receptor erbB1 | P00533 |
|  |  | Steryl-sulfatase | P08842 |
|  |  | Caspase-1 | P29466 |
| 8 | Borapetoside B | Cannabinoid receptor 1 (by homology) | P21554 |
|  |  | TNF-alpha | P01376 |
|  |  | Epidermal growth factor receptor erbB1 | P00533 |
|  |  | Caspase-1 | P29466 |
| 9 | Borapetoside C | Epidermal growth factor receptor erbB1 | P00533 |
|  |  | Steryl-sulfatase | P08842 |
|  |  | Caspase-1 | P29466 |
| 10 | Borapetoside D | Peroxisome proliferator-activated receptor gamma | P37231 |
|  |  | Peroxisome proliferator-activated receptor alpha | Q07869 |
|  |  | Epidermal growth factor receptor erbB1 | P00533 |
|  |  | Caspase-1 | P29466 |
| 11 | Borapetoside E | Peroxisome proliferator-activated receptor gamma | P37231 |
|  |  | Epidermal growth factor receptor erbB1 | P00533 |
|  |  | Caspase-1 | P29466 |
| 12 | Borapetoside F | Epidermal growth factor receptor erbB1 | P00533 |
|  |  | Caspase-1 | P29466 |
| 13 | Cycloeucalenone | Acetyl-CoA carboxylase 4 | O00763 |
|  |  | 11-beta-hydroxysteroid dehydrogenase 1 | P28845 |
|  |  | Cytochrome P450 19A1 | P11511 |
| 14 | Dihydrodiscretamin | PI3-kinase p110-alpha/p85-alpha | P27986 |
|  |  | Insulin receptor | P06213 |
|  |  | Androgen Receptor | P10275 |
|  |  | 1-acylglycerol-3-phosphate O-acyltransferase beta | O15120 |
| 15 | Luteolin 4'-methyl ether 7-glucoside | TNF-alpha | P01379 |
|  |  | Epidermal growth factor receptor erbB1 | P00533 |
|  |  | Caspase-1 | P29466 |
| 16 | Makisterone C | Peroxisome proliferator-activated receptor gamma | P37231 |
|  |  | TNF-alpha | P01377 |
|  |  | Epidermal growth factor receptor erbB1 | P00533 |
|  |  | Serine/threonine-protein kinase AKT2 | P31751 |
|  |  | Androgen Receptor | P10275 |
|  |  | Acetyl-CoA carboxylase 3 | O00763 |
|  |  | 11-beta-hydroxysteroid dehydrogenase 1 | P28845 |
|  |  | Cytochrome P450 19A1 | P11511 |
| 17 | N-acetylanonaine | Epidermal growth factor receptor erbB1 | P00533 |
|  |  | Nitric-oxide synthase, endothelial | P29474 |
|  |  | Cytochrome P450 19A1 | P11511 |
| 18 | N-Acetylnornuciferine | Epidermal growth factor receptor erbB1 | P00533 |
|  |  | Cannabinoid receptor 1 (by homology) | P21554 |
|  |  | Androgen Receptor | P10275 |
|  |  | Cytochrome P450 19A1 | P11511 |
| 19 | N-cis-Feruloyltyramine | Insulin receptor | P06213 |
|  |  | TNF-alpha | P01375 |
|  |  | Epidermal growth factor receptor erbB1 | P00533 |
| 20 | N-Formylanonaine | Cannabinoid receptor 1 (by homology) | P21554 |
|  |  | Nitric-oxide synthase, endothelial | P29474 |
|  |  | Epidermal growth factor receptor erbB1 | P00533 |
|  |  | Cytochrome P450 19A1 | P11511 |
| 21 | N-formylasimilobine 2-O-Beta-D-glucopyranoside | Peroxisome proliferator-activated receptor gamma | P37231 |
|  |  | Peroxisome proliferator-activated receptor alpha | Q07869 |
| 22 | N-trans-caffeoyltyramine | Insulin receptor | P06213 |
|  |  | Epidermal growth factor receptor erbB1 | P00533 |
|  |  | Androgen Receptor | P10275 |
|  |  | Cytochrome P450 19A1 | P11511 |
| 23 | N-trans-Feruloyltyramine | Insulin receptor | P06213 |
|  |  | TNF-alpha | P01378 |
|  |  | Epidermal growth factor receptor erbB1 | P00533 |
|  |  | Serine/threonine-protein kinase AKT2 | P31751 |
| 24 | Paprazine | Insulin receptor | P06213 |
|  |  | Peroxisome proliferator-activated receptor gamma | P37231 |
|  |  | Epidermal growth factor receptor erbB1 | P00533 |
|  |  | Androgen Receptor | P10275 |
| 25 | Rumphioside A | Epidermal growth factor receptor erbB1 | P00533 |
|  |  | Caspase-1 | P29466 |
| 26 | Rumphioside B | Epidermal growth factor receptor erbB1 | P00533 |
|  |  | Caspase-1 | P29466 |
| 27 | Tembetarine | Androgen Receptor | P10275 |
|  |  | Adrenergic receptor beta | P07550 |
| 28 | Tinocrispol A | Epidermal growth factor receptor erbB1 | P00533 |
|  |  | 11-beta-hydroxysteroid dehydrogenase 1 | P28845 |
| 29 | Tinoscorside A | Insulin receptor | P06213 |
|  |  | Epidermal growth factor receptor erbB1 | P00533 |
| 30 | Tyramine | Epidermal growth factor receptor erbB1 | P00533 |
|  |  | Androgen Receptor | P10275 |
|  |  | Adrenergic receptor beta | P07550 |
|  |  | Cytochrome P450 19A1 | P11511 |
