# Supplementary material for: Network pharmacology integrated molecular dynamics reveals the bioactive compounds and potential targets of Tinospora crispa Linn. as insulin sensitizer
Source: PLoS One. 2022 Jun 23;17(6):e0251837. doi: 10.1371/journal.pone.0251837 (PMC9223613; doi:10.1371/journal.pone.0251837)
Supplement: S3 Table — (DOCX) [file pone.0251837.s004.docx]

**S3 Table. Molecular docking result of *T. crispa* phytoconstituents to 7 selected target proteins using AutoDock**

| Protein target | TC Compound | Lowest binding energy | Hidrogen bond | Hydrophobic bond |
| --- | --- | --- | --- | --- |
| PI3K | Tinoscorside A | **-11.64** | Asp96, Lys833 | Ala 805, Thr 887, Met 804, Pro 810, Asp 950. Ile 953, Ile 879, Tyr 867, Met 953, Ile 831, phe 961, Glu 880, Ile 881, Val 882, Trp 812, Lys 890 |
| PI3K | Borapetoside A | -9.88 |  | Asn 951, Ile 963, Lys 833, Lys 807, Asp 964, Ile 831, Val 882, Tyr 867, Glu 880, Ile 879, Ile 881, Met 804, Ser 806, Pro 810 |
| PI3K | *N-formylasimilobine 2-O-Beta-D-glucopyranoside* | -9.65 | Val 883, Lys 890 | Phe 961, Glu 880, Ile 881, Ala 885, Met 953, Ile 879, Tyr 867, Ile 831, Ile 963, Asn 951, Asp 950, Thr 887, Trp 812 |
| PTPN1 | Beta sitosterol | -8.65 |  | Gly 720, Asp 691, Arg 721, Cys 715, Ala 717, Tyr 546, Phe 482, Asp 548, Ser 716, Gln 762, Ile 719, Gly 759, Val 549. |
| PTPN1 | Jatrorrhizine | -7.79 | Arg 721 | Asp 681, Lys 620, Tyr 546, Ser 716, Asp 548, Val 549, Ile 719, Gln 762, Ala 717, Phe 682. |
| PTPN1 | Magnoflorine | -7.71 | Gly 720, Cys 715, Ala 717, Ser 716, Tyr 546, Lys 620. | Arg 721, Ile 719, Phe 682, Asp 548, Gln 762, Asp 681. |
| PPARG | Borapetoside A | -9.94 |  | Arg 280, Ile 281, Met 348, Cys 285, Leu 353, Leu 330, Ser 342, Leu 333, Ile 341, Glu 343, Leu 340, Gly 284, Arg 288, |
| PPARG | Stigmasterol | -9.71 | Arg 280 | Leu 255, Leu 330, Leu 333, Ile 341, Val 339, Met 348, Leu 340, Ser 342, Arg 288, Cys 285, Gly 284, Glu 343, Phe 287, Ile 281. |
| PPARG | Beta sitosterol | -9.65 |  | Lys 263, Ser 342, Phe 287, Gly 284, Arg 288, Cys 285, Ile 281, Leu 353, Met 364, Met 348, Ile 341, Glu 259, Ile 262, Gly 258. |
| INSR | Tinoscorside A | **-11.43** | Asp 1083, Ser 1086, Met 1079. | His 1081, Gly 1003, Ala 1028, Leu 1078, Val 1010, Leu 1002, Met 1139, Glu 1012, Arg 1000, Arg 1026, Gly 1082, Ala 1080. |
| INSR | N-trans-Feruloyltyramine | -8.85 | Ser 1086, Asp 1083, Met 1079. | Ser 1090, His 1081, Leu 1002, Met 1139, Leu 1078, Gly 1082, Tyr 1087. |
| INSR | N-cis-feruloyltyramine | -8.52 | Met 1079, Asp 1083. | Leu 1078, Ala 1080, Arg 1000, Leu 1002, Ser 1086, Tyr 1087, Ser 1090, His 1081, Met 1139, Gly 1082. |
| EGFR | Tinoscorside A | -9.65 | Asp 855,Thr 845, Cys 797. | Leu 792, Pro 794, Leu 844, Met 793, Ala 743, Thr 790, Met 766, Lys 745, Val 726, Asp 800, Leu 718, Gly 796. |
| EGFR | Borapetoside B | -9.48 |  | Pro 794, Leu 718, Met 793, Leu 844, Thr 790, Ala 743, Thr 854, Asp 855, Asn 842, Arg 841, Cys 797, Leu 792, Gly 796. |
| EGFR | Borapetosida A | -9.21 | Thr 854 | Leu 718, Leu 844, Val 726, Met 793, Ala 743, Thr 790, Leu 792, Ile 789, Leu 777, Leu 788, Met 766, Lys 745, Lys 755, Gly 796. |
| TNF | Borapetoside B | -7.9 |  | Ile 155, Leu 57, Gly 121, Leu 120, Tyr 119, Tyr 151, Tyr 59. |
| TNF | Cycloeucalenol | -6.95 | Gln 149 | Tyr 119, Gln 61, Leu 120, Gly 122, Gly 121, Val 123, Leu 57, Tyr 59, Tyr 151. |
| TNF | Borapetoside H | -5.84 |  | Tyr 151, Tyr 59, Tyr 119, Leu 120, Gly 121, Ser 60. |
| AKT2 | Makisterone C | **-11.3** | Tyr 327, Cys 311, Lys 298, Arg 274, His 196. | - |
| AKT2 | Higenamine | -8.63 | Tyr 327, Cys 311, Lys 298, Arg 274, His 196. | - |
| AKT2 | Borapetol A | -8.17 | Tyr 327, Cys 311, Lys 298, Arg 274, His 196. | - |
